# A new defoliating threat to eucalyptus plantations: biology and foliar consumption of *Physocleora dukinfeldia* (Lepidoptera: Geometridae)

**DOI:** 10.7717/peerj.20589

**Published:** 2026-01-12

**Authors:** Paula Gregorini Silva, Aline Marques Pinheiro, Thais Lohaine Braga Santos, Laura Vilas Bôas Gianezi, Daniel Somma Araújo, Bruna Ferreira Anjos, Carlos Gilberto Raetano, Carlos Frederico Wilcken

**Affiliations:** 1 Department of Crop Protection, São Paulo State University, Botucatu, São Paulo, Brazil; 2 Department of Sustainability, Research and Innovation, Suzano S.A, Três Lagoas, Mato Grosso do Sul, Brazil

**Keywords:** Eucalyptus, Forest pest, Insect biology, Host adaptation

## Abstract

The defoliator *Physocleora dukinfeldia* Schaus 1897 (Lepidoptera: Geometridae) was recently reported attacking *Eucalyptus urograndis* (Myrtaceae) plantations in Brazil, raising concerns about its potential impact on commercial forestry. In this context, early characterization of pest biology plays a critical role in understanding host-use patterns and forecasting potential impacts on forest ecosystems. This study aimed to characterize the biology and foliar consumption of *P. dukinfeldia* on *E. urograndis* and its native host, *Schinus terebinthifolia* (Anacardiaceae), by recording molting, mortality, pupation, and emergence of imago, measuring larval head capsule width, pupal weight, and size, and calculating stage duration and viability under controlled conditions. The insect completed its life cycle on both host plants, with significantly longer development time and reduced pupal viability observed on *E. urograndis*. Although larval survival was low on both host species, nearly one-third of the individuals successfully reached adulthood. No significant differences in leaf consumption were detected between the two eucalyptus species evaluated in this study. These findings indicate that *P. dukinfeldia* has the biological capacity to adapt to eucalyptus and should be closely monitored in forest production areas. This is the first report to detail the life cycle and feeding behavior of this species on eucalyptus, providing critical baseline information for future pest management strategies.

## Introduction

Eucalyptus cultivation stands out in the Brazilian forestry sector due to its high economic viability and versatile applications (Barroso et al., 1984). Although the genus *Eucalyptus* comprises approximately 730 species, only about 20 are commercially exploited, mainly to produce paper, pulp, timber, charcoal, essential oils, and biomass (Magaton et al., 2009; Penfold & Willis, 1954). Currently, eucalyptus plantations cover approximately 7.8 million hectares, accounting for 76% of all planted forests in the country (Ibá, 2024).

The expansion of eucalyptus cultivation areas in Brazil has been accompanied by an increase in the occurrence of diseases and phytophagous insects, many of which are recognized as potential pests capable of causing serious damage to the crop (Pereira et al., 2001). The harm caused by these insect pests can compromise forest productivity directly in the field (Guo et al., 2023; Kogan, 1998). Among the main groups responsible for such losses are defoliating caterpillars, which directly impact plant development by causing partial or even complete defoliation of the canopy, an effect that can severely hinder the growth and yield of commercial eucalyptus plantations (Ribeiro et al., 2013; Zanuncio et al., 2018).

Within the Lepidoptera, the family Geometridae includes several genera recognized for their defoliating potential in forest ecosystems. Geometridae larvae commonly feed on woody plants, especially trees and shrubs. Key host plant families include Fabaceae, Rosaceae, Piperaceae, Anacardiaceae, and Bignoniaceae (Bodner et al., 2010; Nunes et al., 2013; Vargas-Ortiz & Parra, 2025). One of the best-known examples is the Neotropical genus *Physocleora*, which occurs throughout much of Central and South America. Its distribution extends from southern Mexico through northern Argentina with more recent records also reported from northernmost Chile (Pitkin, 2002; Rajaei et al., 2022; Vargas-Ortiz & Parra, 2025).

Among defoliating caterpillars, a species that has recently gained attention is *Physocleora dukinfeldia* (Lepidoptera: Geometridae) (Warren, 1897). Originally recorded in Brazil in the municipality of Castro, Paraná, Brazil (Schaus, 1897), this species was only recognized as a pest in 2021, following an outbreak in eucalyptus plantations that resulted in severe defoliation and economic losses. Similar to other geometrid caterpillars known to affect forest crops, *P. dukinfeldia* has demonstrated the potential to become a significant threat to commercial eucalyptus production (Silva et al., 2023; Pereira, 2012).

The emergence of *P. dukinfeldia* as an eucalyptus pest highlights a broader ecological process: the colonization of new host plants by phytophagous insects and frequently precedes the emergence of pest outbreaks in managed ecosystems (Charlery de la Masselière et al., 2017). When a native herbivore shifts to a commercially important plant species, it may find favorable conditions for development due to reduced interspecific competition, lack of natural enemies, or continuous food availability, factors often present in large-scale monocultures. However, successful establishment in a novel host requires physiological and behavioral plasticity, which must be assessed through detailed biological studies (Agrawal, 2000; Schoonhoven, Van Loon & Dicke, 2005).

Cases of geometrid and other insect defoliation of *Eucalyptus* plantations have been extensively documented in Australia, where native taxa sometimes exploit plantation conditions and cause substantial foliage loss. Studies on outbreaking geometrids such as the autumn gum moth (*Mnesampela privata*) and on major eucalypt herbivores more generally have provided important insights into how plantation age, host-plant phenology, and plantation monoculture can promote host-use expansion and episodic outbreaks (Loch & Matsuki, 2010; Rapley, Allen & Potts, 2004; Steinbauer, Mcquillan & Young, 2001). Incorporating these findings into the global context helps frame the significance of newly observed *Eucalyptus* infestations elsewhere and motivates comparative studies of host-shift dynamics in other regions.

Understanding the life cycle parameters of herbivorous insects on new hosts, including development time, survival rates, reproductive capacity, and feeding behavior, is essential to assess their establishment potential and predict future population dynamics (Gripenberg et al., 2010). Such data are crucial in forest production systems, where outbreaks of defoliators can lead to significant reductions in growth and wood yield. Moreover, early-stage studies on insect biology can reveal signs of host-use constraints or adaptive potential, guiding monitoring efforts and informing integrated pest management programs (Heidel-Fischer et al., 2019; Janz, Nylin & Wahlberg, 2006). In this context, this study aimed to evaluate the biological performance of *P. dukinfeldia* on *E. urograndis* compared to its native host, *Schinus terebinthifolia*, to assess this pest’s potential for adaptation and establishment in commercial eucalyptus plantations. For this, were recorded the duration and success of developmental stages (molting, death, pupation, and emergence of imago), measuring morphometric parameters (larval head capsule width, pupal weight, and size), and calculating overall development time and viability.

## Materials and Methods

### *Physocleora dukinfeldia* rearing

*P. dukinfeldia* individuals were initially collected in a commercial eucalyptus area in Guatapará, SP, Brazil. Eggs and larvae were maintained in plastic containers (500 mL) covered with organza mesh, under controlled conditions (25 ± 2 °C, 70 ± 10% relative humidity, and a 12-hour photoperiod). Larvae were fed with pure eucalyptus leaves (*Eucalyptus urophylla*), obtained from healthy plants. The pupae were kept in containers with soil substrate until complete sclerotization. Then, they were transferred to containers lined with moistened filter paper. Adults were kept in polyvinyl chloride (PVC) mesh cages, where a 20% honey solution and brown paper were available for adult feeding and female oviposition, respectively. Papers containing eggs were used to start new rearing cycles.

### Life cycle assessment

The biology of *P. dukinfeldia* was assessed on two host plants, *Eucalyptus urograndis* (*E. grandis* × *E. urophylla*) and *Schinus terebinthifolia* (Brazilian peppertree), to better understand the developmental cycle of this defoliator species. The hybrid *E. urograndis* was selected because it represents the predominant genotype used in Brazilian commercial plantations and corresponds to the host on which *P. dukinfeldia* infestations were first recorded. The tree species used in the experiment were obtained from the Department of Crop Protection, São Paulo State University, Botucatu, SP, Brazil.

Neonate larvae were individually placed in plastic containers (500 mL) containing leaves from each host plant. The leaves were inserted into Eppendorf tubes filled with water to maintain leaf turgor. Each container was considered an experimental unit, with 100 replicates per tree species, in a completely randomized design. The experiment was carried out in a climate-controlled room (25 ± 2 °C, 70 ± 10% relative humidity, and a 12-hour photoperiod).

The insects were inspected daily to measure larval head capsule width, pupal weight (24 h post-pupation), morphological characteristics of the pupae, dates of pupation and imago emergence, and viability in individual periods of development. Based on these records, the following parameters were determined: duration of each larval instar, total larval period, duration of prepupal and pupal stages, total development time (larva-adult), and larval and pupal viability (%).

Head capsules were measured using an ocular micrometer (precision ± 0.001 mm) attached to a stereomicroscope (Leica^®^), with 20 replicates. Pupal weights were obtained with an analytical balance (Shimadzu^®^, model ATY224; accuracy ± 0.01 mg), and pupal length and width were measured with a caliper (precision ± 0.01 mm). During inspection, excrements were removed, and consumed leaves were replaced.

### Leaf consumption

During the development cycle assay, consumed leaves of *E. urograndis* were photographed for subsequent assessment of leaf area consumption. In parallel, an independent assay was conducted using *E. urophylla* leaves to compare foliar consumption between this species and the eucalyptus hybrid (*E. urograndis*). The remaining leaf area after feeding was measured using ImageJ software (Ferreira et al., 2022). Leaves of *S. terebinthifolia* were not included in this analysis due to their morphology, which prevented accurate measurement of leaf area.

### Statistical analysis

Data on developmental time (for each instar, prepupal, and pupal stages), head capsule width, pupal size, and leaf area consumed were subjected to analysis of variance (ANOVA), and the F-test was used to detect significant effects of host plant species. The assumption of residual normality was assessed by the Shapiro–Wilk test, and the homogeneity of variances was verified using Levene’s test (PROC UNIVARIATE; SAS). When significant differences among treatments were detected, the means were compared by the Tukey’s test at a 5% significance level, using the PROC MIXED procedure (SAS Institute, 2011).

## Results

### Life cycle determination

The development of *P. dukinfeldia* occurred through six larval instars on both host plants. Most developmental stages did not differ significantly between *E. urograndis* and *S. terebinthifolia* (Table 1).

**Table 1:** Mean length (±SE) (days) of instar periods, larval period, prepupal period, pupal period and larva-adult development cycle of *Physocleora dukinfeldia* in two forest species.

**Species**	**1** ^**st**^ ** instar**	**2** ^**nd**^ ** instar**	**3** ^**rd**^ ** instar**	**4** ^**th**^ ** instar**	**5** ^**th**^ ** instar**	**6** ^**th**^ ** instar**	**Larval period**
*Eucaliptus urograndis*	4.90 ± 0.17	4.38 ± 0.16	4.97 ± 0.26	5.86 ± 0.23 a	4.97 ± 0.17	11.43 ± 0.39	35.07 ± 0.54 a
*Schinus terebinthifolia*	5.58 ± 0.21	4.00 ± 0.14	4.20 ± 0.19	4.21 ± 0.11 b	5.35 ± 0.21	9.22 ± 0.45	31.56 ± 0.56 b
*P*	0.1054	0.2559	0.1441	0.0002	0.4054	0.0524	0.0180
	**Prepupal period**		**Pupal period**		**Larva-adult period**		
*Eucaliptus urograndis*	2.33 ± 0.12		15.04 ± 0.20		52.00 ± 0.53 a		
*Schinus terebinthifolia*	1.88 ± 0.06		14.38 ± 0.28		48.13 ± 0.60 b		
*P*	0.0755		0.3313		0.0160		

**Notes.**

1 Means followed by the same lowercase letter per column do not differ from each other by the LS-Means adjusted by Tukey’s test (*P* ≤ 0.05).

However, the fourth instar lasted significantly longer on *E. urograndis* (*F* = 15.56; *P* = 0.0002), contributing to an extended total larval period on this host. Consequently, the overall larva-adult development time was also longer on *E. urograndis* than on *S. terebinthifolia* (*F* = 6.18; *P* = 0.0160). No significant differences were observed for the prepupal or pupal stages.

### Cephalic capsule measurements

Head capsule width increased progressively across the six larval instars on both host plants, following the typical geometric growth pattern observed in Lepidoptera. A significant difference between host plants was detected only in the second instar, when larvae fed on *E. urograndis* exhibited slightly larger head capsule widths than those fed on *S. terebinthifolia* (*F* = 5.73; *P* = 0.0217; Table 2). No differences were observed for the remaining instars or between sexes.

**Table 2:** Mean (±SE) head capsule width (mm) of *Physocleora dukinfeldia* larvae fed on two forest tree species.

**Species**	**2** ^**nd**^ ** instar**	**3** ^**rd**^ ** instar**	**4** ^**th**^ ** instar**	**5** ^**th**^ ** instar**	**6** ^**th**^ ** instar**
*Eucaliptus urograndis*	0.28 ± 0.01 a	0.46 ± 0.01	0.74 ± 0.02	1.15 ± 0.03	1.65 ± 0.05
*Schinus terebinthifolia*	0.26 ± 0.00 b	0.43 ± 0.00	0.71 ± 0.01	1.07 ± 0.02	1.53 ± 0.03
*P*	0.0217	0.0583	0.2408	0.0633	0.0574

**Notes.**

1 Means followed by different lowercase letters per column differ from each other by the LS-Means adjusted by Tukey’s test (*P* ≤ 0.05).

### Larval and pupal viability

**Figure 1:**
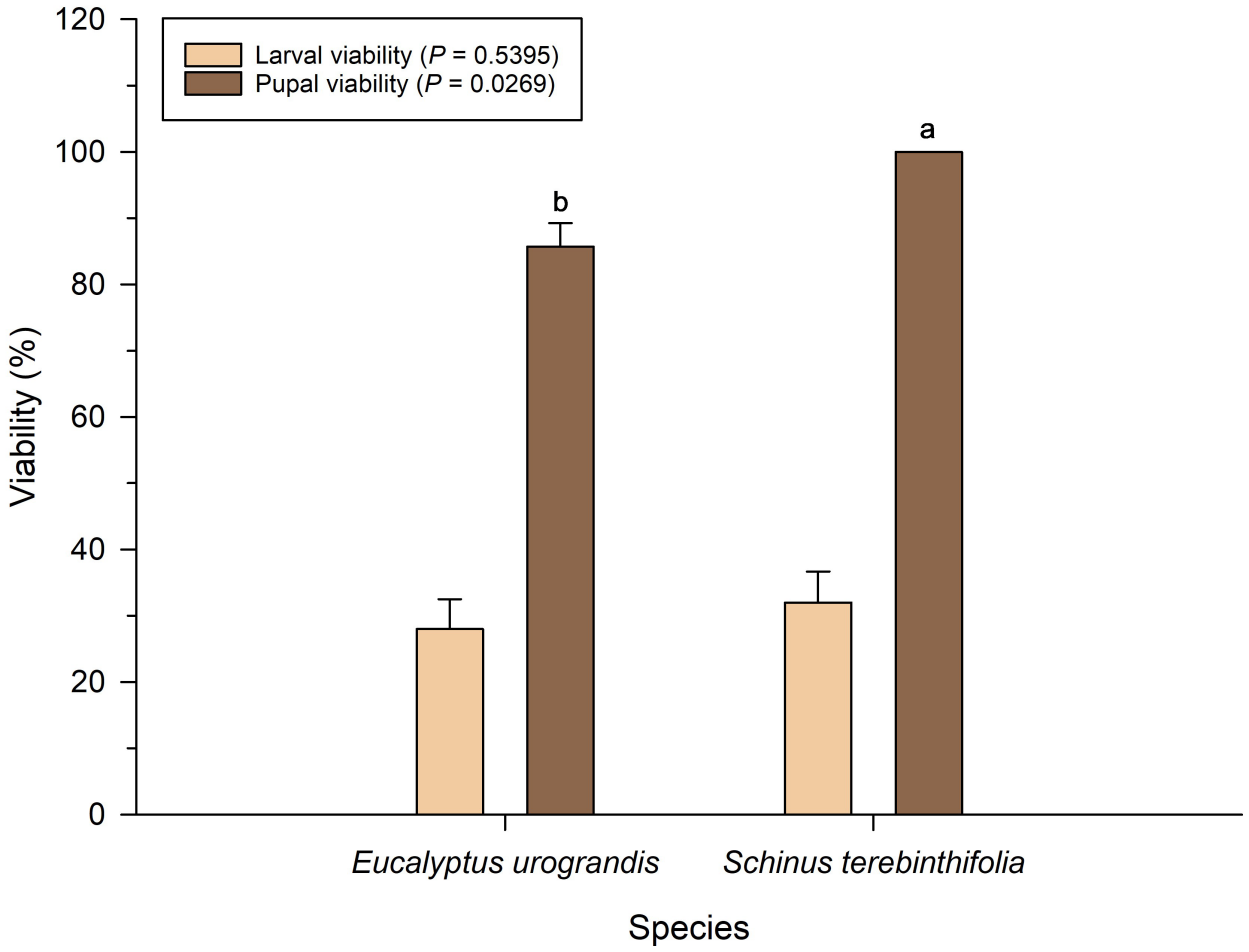
Larval and pupal viability of *Physocleora dukinfeldia* in forest species. Bars represent mean values ± standard error (SE). Means followed by the same lowercase letter do not differ from each other by the Tukey’s test (*P* ≤ 0.05).

Larval viability of *P. dukinfeldia* was low on both host plants, with no statistical difference between treatments (*F* = 0.38; *df* = 1; *P* = 0.5395; Fig. 1). However, pupal viability was significantly lower in larvae fed on *E. urograndis* compared to those reared on *S. terebinthifolia* (*F* = 5.16; *df* = 1; *P* = 0.0269).

The morphological characteristics of *P. dukinfeldia* pupae, including weight, length, and width, showed no statistically significant differences between host plants for either sex (Table 3).

**Table 3:** Mean number (±SE) of weight, length, and width of male and female pupae of *Physocleora dukenfildea* from larvae fed with two forest species.

**Species**	**Weight (mg)**	**Length (mm)**	**Width (mm)**
	**Males**		
*Eucaliptus urograndis*	0.07 ± 0.00	10.90 ± 0.25	3.32 ± 0.06
*Schinus terebinthifolia*	0.07 ± 0.00	11.09 ± 0.13	3.37 ± 0.06
*P*	0.085	0.53	0.67
	**Females**		
*Eucaliptus urograndis*	0.0747 ± 0.00	11.57 ± 0.12	3.44 ± 0.04
*Schinus terebinthifolia*	0.0863 ± 0.00	11.77 ± 0.15	3.56 ± 0.05
*P*	0.076	0.37	0.18

### Foliar consumption

**Figure 2:**
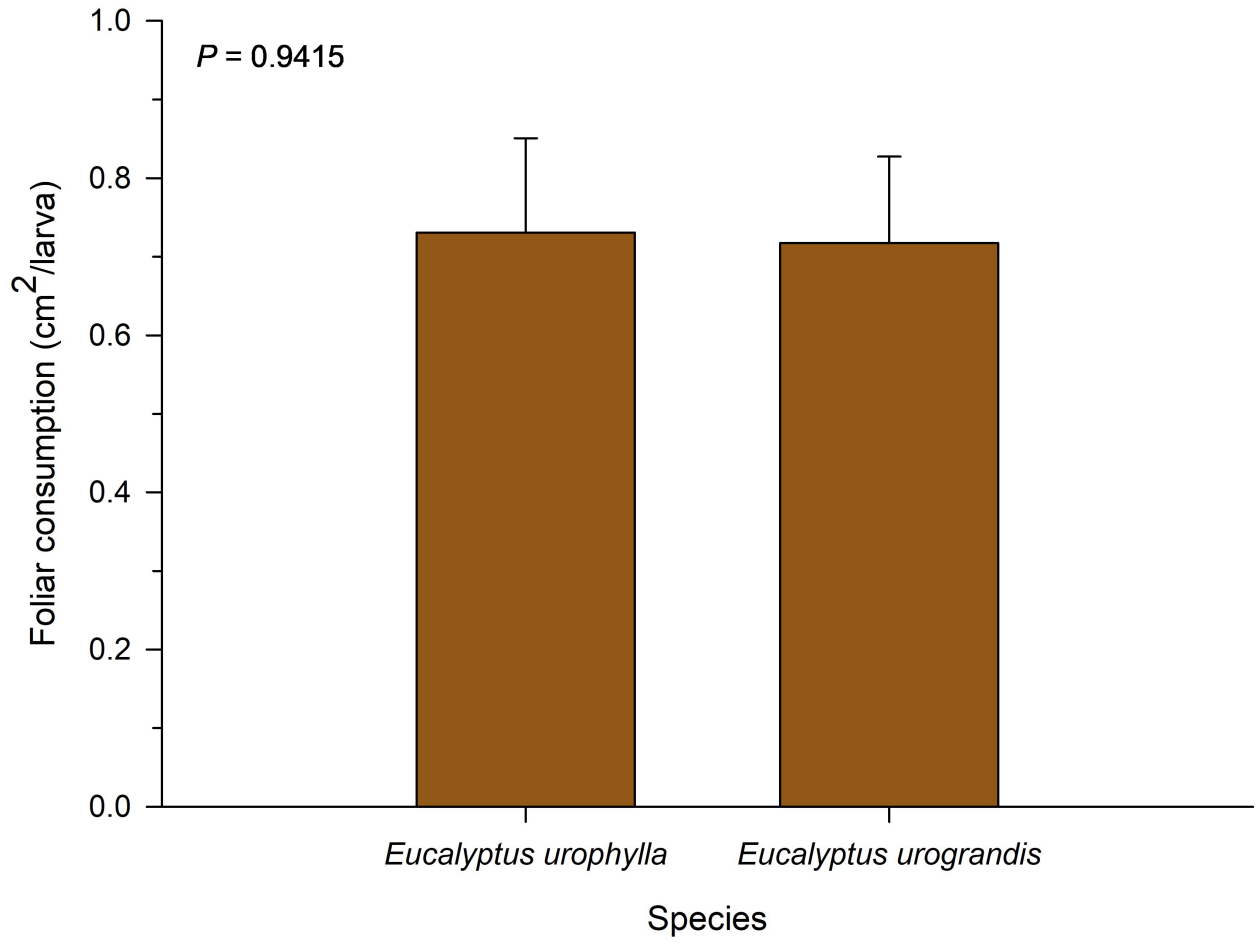
Total leaf consumption of *Physocleora dukinfeldia* larvae fed with leaves of host species. Bars represent mean values ± SE. This parameter considers the total consumed by the individual until its death or passage to the prepupal phase.

No significant differences were observed for total leaf consumption among the eucalyptus species evaluated in this study (*F* = 0.01; *df* = 1; *P* = 0.9514; Fig. 2). The consumption of *P. dukinfeldia* was 0.7309 cm^2^/larva and 0.7173 cm^2^/larva in *E. urophylla* and *E. urograndis*, respectively.

## Discussion

The results demonstrated that *P. dukinfeldia* is capable of completing its life cycle on *E. urograndis*, although with a significantly prolonged larval development time and life cycle (larva-adult) compared to its native host, *S. terebinthifolia*. The observed extension, particularly during the fourth instar and the total larval period, suggests that eucalyptus may impose physiological or nutritional challenges to the development of this species, which is consistent with patterns reported for other insect herbivores (Campos et al., 2022).

The increase in developmental time from the fourth instar onward may indicate a delayed adaptive response to secondary compounds present in *E. urograndis*, such as monoterpenes and sesquiterpenes, which are commonly associated with the chemical defense of *Eucalyptus* species (Araújo et al., 2010; Cheng et al., 2009; Elangovan & Mudgil, 2023). However, the ability to complete development on *E. urograndis* demonstrates that *P. dukinfeldia* has the potential to establish and adapt to this crop, which justifies special attention in monitoring its occurrence in commercial eucalyptus plantations. Furthermore, the longer life cycle observed on the eucalyptus hybrid (52 days) is consistent with that of other defoliator species, such as *Thyrinteina arnobia* (Stoll, 1782) and *Iridopsis panopla* (Prout, 1932), whose developmental duration may also range from 40 to 60 days depending on environmental conditions and host plant (Jesus et al., 2015; Oliveira et al., 2005; Santos et al., 1996).

The mean head capsule width of *P. dukinfeldia* increased progressively across the six instars on both host plant species, reflecting the typical larval growth pattern observed in Lepidoptera. The consistent increment in head capsule width throughout the instars indicates a stable growth rate, possibly associated with the larva’s physiological adaptation to the host plant (Calvo & Molina, 2008; Castañeda-Vildózola et al., 2016; Panzavolta, 2007).

The low survival rate during the larval stage may reflect the species’ intrinsic sensitivity to host plant defenses (Araújo et al., 2010; Carvalho et al., 2024). However, nearly one-third of the larvae successfully completed development, demonstrating the pest’s potential to establish in commercial eucalyptus plantations. Pupal viability was significantly lower in larvae fed on *E. urograndis*, suggesting that sublethal effects of feeding on the alternative host may accumulate and impact the final transition to adulthood. This reduction in performance is characteristic of early stages of host range expansion, during which the pest is still undergoing adaptation to the novel host (Agrawal, 2003). Over time, *P. dukinfeldia* populations may experience selection and adapt to eucalyptus, potentially increasing host-use efficiency and reducing observed mortality rates.

Despite the variations in development time and viability rates observed between host plants, *P. dukinfeldia* maintained a stable phenotypic pattern during the pupal stage. The absence of morphometric differences also suggests that although *E. urograndis* may pose challenges during larval development, the surviving individuals do not exhibit visible impairments in final growth. This pattern is common in cases of compensatory ontogenetic selection, in which individuals with greater ability to utilize the host offset early developmental deficits with more efficient growth rates during the later stages (Maino & Kearney, 2015). Furthermore, pupal size is directly related to the reproductive potential of adults, especially females, with direct implications for fecundity and population viability (Lee et al., 2023; Santos et al., 2023).

Our observation that *P. dukinfeldia* can complete development on *Eucalyptus* under controlled conditions echoes patterns reported for Australian geometrids and other eucalypt herbivores. In general, outbreaks are often associated with tree-growth season after planting, a simplified plantation structure that reduces spatial heterogeneity, as well as the distribution of natural enemies (Azevedo-Schmidt et al., 2023; Schröder et al., 2021; Steinbauer, 2005; Steinbauer et al., 2004). Thus, the prolonged development and reduced pupal viability we observed on *E. urograndis* are consistent with an early stage of host-use expansion, rather than immediate high-fitness adaptation, and mirror the demographic and ecological mechanisms described for outbreaking eucalypt defoliators. These parallels suggest that similar plantation-scale drivers (monoculture, host availability and phenology, and altered predator-parasitoid communities) should be considered when forecasting the potential for *P. dukinfeldia* to become a recurrent pest in Brazilian eucalyptus stands.

In addition, although *S. terebinthifolia* (Anacardiaceae) and *E. urograndis* (Myrtaceae) are taxonomically distant, both families contain aromatic species rich in terpenoids and phenolic compounds (references), which could partially overlap in chemical cues used by herbivores during host recognition or detoxification. Such chemical convergence has been proposed as an important factor in facilitating host shifts across unrelated plant taxa (Nylin & Janz, 2009; Nylin, Slove & Janz, 2014). Generalist ancestry or latent plasticity in host-use traits may enable herbivorous insects to explore novel hosts when ecological conditions favor such transitions (Nylin & Janz, 2009).

From a nutritional standpoint, both eucalyptus species tested in this study provide similar conditions for leaf consumption by the pest. *P. dukinfeldia* appears to feed with comparable efficiency across different eucalyptus species, reinforcing its potential generalist behavior regarding host plant use. Although individual foliar consumption was relatively low in this study (less than one cm^2^ throughout development), this may be offset by population outbreaks if environmental conditions favor pest density increases. Therefore, even species with low individual consumption can cause significant damage at the population level, particularly during the early stages of infestation and in the absence of effective management strategies (Speight, Hunter & Watt, 2008).

## Conclusion

The emergence of *P. dukinfeldia* as a defoliator of *E. urograndis* highlights the need for proactive biological studies on newly observed pest species in forest plantations. Although this insect exhibited extended development times and reduced viability on eucalyptus compared to its native host, *S. terebinthifolia*, it was capable of completing its life cycle, indicating its potential to establish and adapt to commercial eucalyptus systems. This pioneering study provides essential baseline data on the biology, development, and feeding behavior of *P. dukinfeldia* under controlled conditions. The results underscore the pest’s capacity to exploit eucalyptus as a viable host, even in the early stages of host adaptation. Given that individual foliar consumption was relatively low, but survival to adulthood was achievable, future outbreaks cannot be ruled out. The data generated here serve as a valuable reference for integrated pest management strategies and reinforce the importance of continuous monitoring and research in the face of emerging defoliators in commercial plantations.

##  Supplemental Information

10.7717/peerj.20589/supp-1 Supplemental Information 1 Raw data for all parameters evaluated

## Additional Information and Declarations

### Data Availability

The following information was supplied regarding data availability: The raw data is available in the Supplemental Files.
